# Neural representational similarity between L1 and L2 in spoken and written language processing

**DOI:** 10.1002/hbm.25171

**Published:** 2020-08-21

**Authors:** Say Young Kim, Lanfang Liu, Li Liu, Fan Cao

**Affiliations:** ^1^ Department of English Language and Literature Hanyang University Seoul Korea; ^2^ Hanyang Institute for Phonetics and Cognitive Sciences of Language, Hanyang University Seoul Korea; ^3^ Department of Psychology Sun Yat‐Sen University Guangzhou China; ^4^ State Key Laboratory of Cognitive Neuroscience and Learning & IDG/McGovern Institute for Brain Research Beijing Normal University Beijing China; ^5^ Center for Collaboration and Innovation in Brain and Learning Sciences Beijing Normal University Beijing China

**Keywords:** bi/multilingual, fMRI, Korean, spoken word processing, visual word processing

## Abstract

Despite substantial research on the brain mechanisms of L1 and L2 processing in bilinguals, it is still unknown whether language modality (i.e., visual vs. auditory) plays a role in determining whether L1 and L2 are processed similarly. Therefore, we examined the neural representational similarity in neural networks between L1 and L2 in spoken and written word processing in Korean–English–Chinese trilinguals. Participants performed both visual and auditory rhyming judgments in the three languages: Korean, English, and Chinese. The results showed greater similarity among the three languages in the auditory modality than in the visual modality, suggesting more differentiated networks for written word processing in the three languages than spoken word processing. In addition, there was less similarity between spoken and written word processing in L1 than the L2s, suggesting a more specialized network for each modality in L1 than L2s. Finally, the similarity between the two L2s (i.e., Chinese and English) was greater than that between each L2 and L1 after task performance was regressed out, especially in the visual modality, suggesting that L2s are processed similarly. These findings provide important insights about spoken and written language processing in the bilingual brain.

## Graphical abstract

Korean–Chinese–English trilinguals showed more differentiated brain network for written word processing than spoken word processing. A more specialized network for auditory and visual modalities in L1 than in L2s. L2s are processed more similarly than L1 and each of L2s, especially in the visual modality.

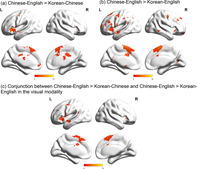

## 1 INTRODUCTION

It has long been questioned whether the brain network for the first (L1) and second (L2) language is shared or separate. Although a substantial number of neuroimaging studies have shown that similar brain regions are involved in L1 and L2 (e.g., Buchweitz, Shinkareva, Mason, Mitchell, & Just, 2012; Cao, Tao, Liu, Perfetti, & Booth, 2013; Kim et al., 2016; Mei, Lu, He, & Chen, 2015; Van de Putte, Baene, Price, & Duyck, 2018), other studies have highlighted differences in brain activation between L1 and L2 (e.g., Jamal, Piche, Napoliello, Perfetti, & Eden, 2012; Li et al., 2019; Tham et al., 2005; Xu, Baldauf, Chang, Desimone, & Tan, 2017). For example, using multivoxel pattern analysis (MVPA), Xu et al. (2017) demonstrated that L1 and L2 elicit brain activation in common regions, but with notably distinguishable patterns in Chinese‐English bilinguals, suggesting that the same brain areas but functionally independent neural populations are involved in L1 and L2. MVPA or other multivariate approaches, such as representational similarity analysis (RSA), analyze the activation *patterns* of multivoxels, reflecting unique representational information (Kriegeskorte, Mur, & Bandettini, 2008) and thereby providing a precise estimation of distributional representation patterns underlying a certain cognitive computation (Wang et al., 2018). Thus, depicting the brain activation patterns of multiple voxels, sheds new light on understanding how similarly or differently L1 and L2 are represented and processed in the bilingual brain.

The previous literature has focused on either written (Liu, Dunlap, Fiez, & Perfetti, 2007; Tan et al., 2003) or spoken (Perani et al., 1998; Saur et al., 2009; Tham et al., 2005) language processing when investigating the relationship between L1 and L2 in the brain. The separate research on written and spoken languages reached a similar conclusion: L1 and L2 are processed in a generally shared network with some accommodations for the specific language features and language proficiency or the age of acquisition (AOA). However, in order to gain a more complete picture of the bilingual brain network, it is important to compare whether the relationship between L1 and L2 is the same in spoken language processing as in written language processing. Written language processing could be expected to be more different across languages than spoken language processing due to the diverse scripts and mapping rules from script to sound/meaning in each language (Perfetti, 2003; Perfetti, Cao, & Booth, 2013; Perfetti & Harris, 2013; Seidenberg, 2011). From the evolutionary viewpoint, for instance, the *neuronal recycling hypothesis* (Dehaene & Cohen, 2007) and the *neuroemergentism* (Hernandez et al., 2019) propose that culturally new inventions such as reading/writing require the involvement of brain regions that are initially responsible for relevant functions, such as face recognition. Thus, it assumes that the reconfiguration of brain mechanisms for reading is a language‐independent mechanism.

Substantial evidence indicates that the reading network varies according to the features of the language. For example, a cross‐linguistic study (Paulesu et al., 2000) has found that deep orthographies such as English are associated with greater activation in the inferior frontal gyrus than shallow orthographies, whereas shallow orthographies such as Italian are associated with greater activation in the temporo‐parietal areas than deep orthographies (see also Jobard, Crivello, & Tzourio‐Mazoyer, 2003). For the deep orthographies, because the grapheme‐to‐phoneme conversion (GPC) is not entirely regular, readers tend to use the whole‐word strategy, whereas for the shallow orthographies, readers likely opt for the assembly strategy, as the GPC is reliable. Reading in Chinese and English involves different brain regions as well. Previous meta‐analyses conclude that, comparatively, reading English is associated with greater activation in posterior regions of the left superior temporal gyrus, whereas reading Chinese elicits greater activation in the left middle frontal gyrus, bilateral temporo‐occipital regions, and the left inferior parietal lobule (Bolger, Perfetti, & Schneider, 2005; Tan, Laird, Li, & Fox, 2005). This might be due to the whole‐character‐to‐whole‐syllable mapping and complex visuo‐orthography in Chinese. As for Korean, the Korean‐related brain network overlaps with that of English reading, but reading Korean seems to elicit more activation in the bilateral middle occipital gyri and left inferior frontal gyrus than reading English (Kim et al., 2016). This might be because of the complex visual forms of Korean. In addition, compared with reading Chinese, reading Korean showed more activation in regions typically involved in phonological processing, including the left inferior parietal lobule, right inferior frontal gyrus, and right superior temporal gyrus (Kim, Liu, & Cao, 2017). These differences in brain activations while reading Korean, English, and Chinese can be explained by the language features. Namely, Chinese is morpho‐syllabic and does not have GPC; the whole character is mapped to the whole syllable. Substantial homophones also encourage the direct mapping between orthography and semantics. English is alphabetic and thus maintains an intimate connection between orthography and phonology. Korean is similar to English in that it is alphabetic, however it is more regular, and its visual form is a nonlinear arrangement of Hangul script, which has a visual layout similar to Chinese (Kim et al., 2016). Thus, previous studies have well documented that neural processing of written languages is associated with language‐specific brain regions, in addition to some overlapping regions.

In contrast to written languages, spoken languages share the principle of mapping speech sounds to meanings, which is the symbolic nature of human language. In addition, spoken language processing has significance in human evolution and exhibits shared genes and brain mechanisms across all languages. Therefore, a speech production and perception pathway exists that involves similar networks across languages (Rueckl et al., 2015). However, it could very well be that differences in phonology and tonal information cause diverse processing in different languages.

Very few studies have examined whether L1 and L2 share a more overlapped brain network in spoken language processing than written language processing (Marian et al., 2007; Van de Putte et al., 2018). Marian et al. (2007) examined which brain areas were involved when late Russian‐English bilingual participants passively viewed or listened to words or nonwords in their L1 and L2. The results showed that L1 and L2 elicited similar cortical networks regardless of their modalities with some variations in the location of activation centers for L1 and L2 within the left inferior frontal gyrus (anterior part for L1 and posterior part for L2) during lexical processing (Marian et al., 2007). Other studies using MVPA, also demonstrated that brain activity was similar during semantic access in L1 and L2, regardless of modalities, in Dutch‐French bilinguals (Van de Putte et al., 2018) and Portuguese‐English bilinguals (Buchweitz et al., 2012). However, none of those studies has directly compared spoken and written language, and the languages under study were all alphabetic (Russian and English or Dutch and French). Therefore, the present study was designed to examine whether there is greater language similarity between L1 and L2 in spoken word processing than in written word processing. Directly comparing L1 Korean and L2s English and Chinese, we used a rhyming judgment task in both the visual and auditory modalities, because we attempted to understand dynamics among languages during phonological processing in bilinguals. The rhyming task has been used to directly examine phonological decoding ability in various populations previously (e.g., Booth et al., 2004; Cao et al., 2013; Kim et al., 2016).

In L1, spoken and written word processing actually show differentiation with some limited overlap in the left inferior frontal gyrus and superior temporal gyrus (Regev, Honey, Simony, & Hasson, 2013). Moreover, the degree of overlap between spoken and written word processing appears to be skill sensitive. Previous neuroimaging studies have shown that adults showed less overlap in brain activation between visual and auditory tasks than did children (Booth et al., 2002a, 2002b; Liu et al., 2008), suggesting a higher degree of modality specialization in adults than in children during word processing. Adults showed greater activation in the left fusiform gyrus for the visual modality than the auditory modality and greater activation in the superior temporal gyrus for the auditory modality than the visual modality (Booth et al., 2002a). Consistent findings are shown in research on language learning. Higher proficiency appears to be characterized by greater specialization, whereas beginning learners tend to use a more diffused network (Wong, Perrachione, & Parrish, 2007). Based on these previous findings, one would expect reduced overlap between the visual and auditory modalities in L1 than L2, because higher proficiency is associated with a more specialized/focused network for specific types of stimuli/calculation. However, the bilingual literature has not investigated the differences between L1 and L2 in terms of modality specialization. In this study of a trilingual group with two different L2s (Chinese and English), we expected to find reduced similarity between visual and auditory tasks in L1 (Korean) than in either L2 (English and Chinese).

There has been a consensus in bilingual research that the brain networks of L1 and L2 are shared at some extent under the influence of several factors, such as AOA (Kim, Relkin, Lee, & Hirsch, 1997), proficiency (Cao et al., 2013; Gao et al., 2017), experience (Tu et al., 2015), and similarity of the writing system (Kim et al., 2016). For instance, increasing proficiency in L2 leads to greater similarity to the native brain network in various bilingual groups, such as Chinese‐English (Cao et al., 2013), English‐German (Stein et al., 2009), Italian‐English (Perani et al., 1998), and French‐English (Golestani et al., 2006). As Kim et al. (2016) shows, the L2 that shares greater similarity with L1 in the mapping principle between orthography and phonology showed greater similarity to L1 in brain activation, suggesting that the similarity of brain networks for L1 and L2 depends on the similarity of their writing systems. Thus, the results of greater similarity between Korean and English than between Korean and Chinese can be interpreted in light of the similarity between English and Korean. Both are alphabetic, whereas Chinese is nonalphabetic. However, those authors compared only the similarity in brain activation between the L1 and L2, not the similarity between the two L2s. It might be that the two L2s are processed more similarly to each other than either is to L1 when proficiency is regressed out, which could suggest that L2s are processed in a qualitatively different way from L1. Therefore, the present study is designed to examine the similarity in brain activity not only between L1 and each of the L2s, but also between the two L2s using a multivariate approach.

The present study recruited trilingual participants who learned two typologically different L2s (Chinese and English) in addition to their L1 (Korean). We used RSA, because it allows us to compare the similarity of activity patterns between conditions, whereas the classifier‐based MVPA aims to distinguish brain activity patterns between conditions. Using the RSA, therefore, we tested whether there is a greater similarity among the three languages in the auditory modality than in the visual modality, whether there is a reduced similarity between the visual and auditory modality for the L1 than for the two L2s, and whether the similarity between the two L2s is greater than that between each L2 and L1. We expected greater similarity between languages for the auditory task than for the visual task because written word processing has more cross‐linguistic differences than spoken word processing. This would be consistent with the idea that written language is a cultural invention that allows cultural variability in the form it takes (Dehaene & Cohen, 2007; Hernandez et al., 2019), whereas speech perception and production play significant roles in human evolution, leading to the development of specifically dedicated genes and brain mechanisms. We hypothesized reduced similarity between the visual and auditory modality for the L1 than for the L2s because higher proficiency is associated with greater modality specialization. In addition, we expected greater similarity between the two L2s than that between the L1 and the L2s, if a language is processed essentially in a different way as long as it is acquired after the first language. If the similarity is determined by language difference no matter whether it is L1 or L2, we would find the similarity between Korean and English to be greater than that between Korean and Chinese, and that between English and Chinese.

## 2 METHODS

### 2.1 Participants

Twenty‐two native Korean speakers who learned English and Chinese as second languages (15 females; mean age = 21.5 years, *SD* = 1.8) were recruited from Beijing. All participants were undergraduate or graduate students in universities in Beijing. We originally recruited 31 participants, but eight of them were subsequently excluded due to their head movement and one was excluded due to extremely low accuracy on the tasks. All participants were right‐handed, free of any neurological disease or psychiatric disorders, did not suffer from attention deficit hyperactivity disorder, and did not have any learning disabilities. Ethics approval was obtained from Beijing Normal University and Michigan State University. Informed consent was obtained from all participants.

### 2.2 Language proficiency and AOA in the L2s

All participants responded on the language background questionnaire that Korean is their first and dominant language. Both English and Chinese proficiency levels were assessed with a word reading test and a reading fluency test (a sentence reading comprehension) in each language (Woodcock, McGrew, & Mather, 2001 for English; Xue, Shu, Li, Li, & Tian, 2013 for Chinese). The scores were transformed into age‐equivalent scores shown in Table 1 (for the Chinese tests, we used an in‐house norm developed by Xue, and Shu, Beijing Normal University, to calculate age‐equivalent scores). Word identification (ID) was marginally significantly higher in Chinese than in English [*t*(21) = 1.993, *p* = .059], and the difference in reading fluency between the L2s was not significant [*t*(21) = 1.255, *p* = .216]. The participants also reported that their proficiency in Chinese was higher than that in English for all three domains (reading, speaking, and listening). Therefore, their proficiency level in Chinese tended to be higher than their proficiency in English. The AOA for English was 8.3, which is significantly earlier than their AOA for Chinese, 14.4 (Table 1).

**TABLE 1:** Language profiles (LEAP‐Q) and scores on the proficiency tests for L2s in the Korean trilingual participants

	Chinese	English	Statistical test (|*t|*)
AOA (years)	14.4 (2.9)	8.3 (2.3)	10.099***
AOA—reading (years)	15.1 (2.8)	10 (2.3)	7.970***
AOF (years)	17 (3.2)	16.9 (3.2)	.134 [N.S]
AOF—reading (years)	16.9 (3.2)	15.1 (2.8)	2.337 *
LOR—country (years)	5.9 (3.0)	0.4 (1.0)	7.775***
Speaking rating (0–10)	7.2 (1.4)	5.0 (2.0)	3.788**
Listening rating (0–10)	7.7 (1.1)	6.3 (1.6)	3.586 **
Reading rating (0–10)	7.5 (1.0)	6.4 (1.9)	2.242*
*Proficiency tests*			
Word ID—raw score	86.2 (29.5)	40.3 (7.5)	
Word ID—age equivalent	9.0 (1.6)	8.4 (.9)	1.993 [N.S]
Reading fluency—raw score	47.5 (13.6)	50.2 (15.2)	
Reading fluency—age equivalent	9.3 (1.7)	10.1 (2.1)	1.255 [N.S]

*Note:* Standard deviations are indicated in parentheses; Speaking rating, listening rating and reading rating were self‐report, with 0 being the lowest and 10 being the highest; English proficiency tests were from WJ‐III tests, and Chinese proficiency tests were Word ID and reading fluency developed by Xue et al. (2013).

Abbreviations: AOA, age of acquisition; AOF, age of fluency; LOR, length of residence; N.S., not significant.

^*^ *p* < .05.

^**^ *p* < .01.

^***^ *p* < .001.

### 2.3 Tasks

During functional magnetic resonance imaging (fMRI), both a visual and an auditory rhyming judgment task using sequentially presented word pairs were presented in each of the three languages (Korean, Chinese, or English), mixed with perceptual control and baseline trials (Table 2 presents examples of the stimuli). The order of the three languages in the visual and auditory modalities was counterbalanced across participants. For each lexical trial, the participant was instructed that he/she would see or hear word pairs one at a time and should decide as quickly and accurately as possible whether the two words rhymed or not, using their right index finger for “yes” and their right middle finger for “no.”

**TABLE 2:** Examples of stimuli in each condition across the three languages

	Language		
Condition	Korean	English	Chinese
O + P+	화분 /hwabun/ ‐ 교문 /kyomun/	Late‐hate	弥补 /mi2bu3/, 纯朴 /chun2pu3/
O + P−	N/A	Pint‐mint	翻译 /fan1yi4/, 选择 /xuan3ze2/
O − P+	정답 /tsʌŋdap/ ‐ 술값 /sulkap/	Jazz‐has	环保 /huan2bao3/, 大炮 /da4pao4/
O − P−	신발 /sinbal/ ‐ 영혼 /yʌŋhon/	Press‐list	损坏 /sun3huai4/, 学科 /xue2ke1/

Abbreviations: O + P+, similar orthography and phonology; O + P−, similar orthography and different phonology; O − P+, different orthography and similar phonology; O − P−, different orthography and phonology.

For the visual rhyming task, each stimulus in each trial was presented for 800 ms, with a 200 ms blank interval between stimuli. For the auditory rhyming task, the duration of each word was between 500 and 800 ms, with a 200 ms blank interval between stimuli, and those auditory stimuli were presented through an MR‐compatible headphone (Optoactive 2 from Optoacoustics). A red fixation cross appeared on the screen immediately after the offset of the second stimulus, indicating the need to make a response. The duration of the red fixation varied (2,200, 2,600, or 3,000 ms), such that each trial lasted for 4,000, 4,400, or 4,800 ms. For the resting baseline trials (*N* = 48), the participant was required to press the “yes” button when a black fixation cross in the center of the screen turned red. Perceptual control trials (*N* = 24) were also included as part of a larger study, but they are not of interest in the present experiment. During the visual perceptual trials, participants were required to indicate whether two sequentially presented symbol patterns were identical or not by pressing the “yes” or “no” button. During the auditory perceptual trials, participants were required to indicate whether two sequentially presented tones were identical or not by pressing the “yes” or “no” button. The timing for the perceptual control and resting baseline trials was the same as for the lexical trials. The order of presentation for the lexical, perceptual, and resting baseline trials and the variation of the response intervals were optimized for event‐related designs by OptSeq (http://surfer.nmr.mgh.harvard.edu/optseq). All participants participated in a 5‐min practice session out of the scanner to get familiarized with the task procedures.

The English and Chinese rhyming judgment tasks used two rhyming and two nonrhyming conditions with 24 trials per condition. As shown in Table 2, one of the two rhyming conditions had similar orthographic and phonological endings (O + P+), and the other had different orthographic but similar phonological endings (O − P+). In two nonrhyming conditions, one had similar orthographic but different phonological endings (O + P−), and the other had different orthographic and phonological endings (O − P−). All English words were monosyllabic without homophones, and they were matched across conditions for written word frequency [*F*_(3,153)_ = 1.087, *p* = .356] and the sum of their written bigram frequency [*F*_(3,188)_ = 1.273, *p* = .285] (English Lexicon Project, http://elexicon.wustl.edu). All Chinese words consisted of two characters and did not have homophones at the word level. Similar orthography was defined as the same phonetic radical for the second character of the word. In half of the trials of the four lexical conditions (rhyming and nonrhyming), the second character of the words had the same tone, and in the other half, they had different tones. The two‐character words and the second character of those words were matched on adult written frequency (Modern Chinese Word Frequency Corpus, 1990) and number of strokes across conditions [*F*_(3,182)_ = 0.589, *p* = .623 for frequency, *F*_(3,188)_ = 1.954, *p* = .122 for stroke].

The Korean task had 24 trials in each of only three conditions, two rhyming and one nonrhyming, namely O + P+, O − P+, and O − P−, because the O + P− condition is not possible in Korean based on its transparent writing system. All Korean words were disyllabic without homographs or homophones at the word level. The written frequency of the words was matched [*F*_(2,136)_ = .019, *p* = .981] across conditions according to the Korean Word Database (2003), Sejong corpus. In addition, word frequency in all three languages was matched [*F*_(2,516)_ = 2.158, *p* = .117].

### 2.4 MRI data acquisition

All MRI images were acquired on a 3.0 Tesla Siemens scanner (Siemens Healthcare, Erlangen, Germany) at Beijing Normal University. The participants lay in the scanner with their head position secured with foam padding. An optical response box was placed in each participant's dominant right hand, and a compression alarm ball was placed in the left hand. The head coil was positioned over the head so that the participant could effectively use a mirror to view the projection screen at the rear of the scanner. Gradient echo localizer images were acquired to determine the placement of the functional slices. For the functional images, a susceptibility weighted single‐shot echo planar imaging (EPI) method with blood oxygenation level‐dependency (BOLD) was used with the following scan parameters: TR = 2000 ms, TE = 20 ms, flip angle = 80°, matrix size = 128 × 128, field of view = 220 × 220 mm, slice thickness = 3 mm (0.48 gap), number of slices = 32 (interleaved). These parameters resulted in a 1.7 × 1.7 × 3.48 mm voxel size. A high resolution, T1 weighted 3D image was also acquired using MP RAGE with the following parameters: TR = 2,300 ms, TE = 3.36 ms, flip angle = 9°, matrix size = 256 × 256, field of view = 256 × 256 mm, slice thickness = 1 mm, number of slices = 160, resulting voxel size = 1 × 1 × 1 mm. The acquisition of the anatomical scan took approximately 9 min and the fMRI scan for each run was 6 min and 44 s for the Chinese and English task and 4 min and 58 s for the Korean task. There were two runs for each task. The order of the six tasks (i.e., 2 modality × 3 language) was counter‐balanced across participants with each participant carrying out three tasks per day for 2 days.

### 2.5 Image analysis

Data analysis was performed using DPARF (Yan & Zhang, 2010; http://rmfri.org/DPARSF) and SPM12 (Statistical Parametric Mapping; http://www.fil.ion.ucl.ac.uk/spm). The following steps were used for data preprocessing: (1) Slice timing correction for interleaved acquisition using sinc interpolation, (2) fourth degree b‐splice interpolation for realignment to the first volume, (3) Trilinear coregistration with the anatomical image, (4) Segmentation of the anatomical image, (5) Normalization of all brains to the standard T1 Montreal Neurological Institute (MNI) adult template with a voxel size = 3 × 3 × 3 mm (12 linear affine parameters for brain size and position, 8 nonlinear iterations and nonlinear basis functions). No smoothing was applied to the images. Participants with greater than 3 mm or 3° of movement for any task were excluded from the study.

The preprocessed images for each participant were analyzed using the general linear model (GLM). Each condition was modeled as a unique regressor, and six head‐motion parameters were modeled as covariates. For each task, the effect of rhyming trials (including O + P+ and O − P+) versus baseline trials (the fixation) was assessed using a one sample *t*‐test. We included only the rhyming conditions because responses to the nonrhyming conditions could be made based on whole syllable comparisons rather than rhyming judgment, and because there were fewer nonrhyming trials in Korean than in Chinese and English. From the GLM analysis, we obtained six *t*‐statistic maps for each participant (visual and auditory modalities in Korean, English, and Chinese). These maps were then submitted into the RSA.

### 2.6 Representational similarity analysis

A whole‐brain searchlight method was applied to calculate the representational similarity across tasks. At each voxel, a sphere ROI containing 125 voxels centered on that voxel was generated. For each language, Pearson's correlations were calculated on the activation patterns within that ROI between the visual and auditory conditions (modality‐similarity), with the accuracy difference between the two modalities as a covariate. The correlation value was assigned to the center of that ROI to represent the similarity in the brain response pattern between visual and auditory rhyming. Pattern similarity (PS) measures the degree of similarity between two patterns, computed as the Pearson's correlation between two feature vectors (i.e., the *t*‐values of voxels in the searchlight). The resulting pattern similarity (PS) maps (i.e., the Pearson correlation coefficient for each voxel) were entered into the second‐level analysis in SPM 12 for group statistics using *t*‐tests. We calculated the PS between languages (language‐similarity) separately for the visual and auditory modalities with the accuracy difference between the two languages as a covariate. Using a series of paired *t*‐tests, we then compared the language‐similarity between the visual and auditory modalities to see whether there is a greater language‐similarity in one modality than the other. In addition, we conducted a conjunction analysis on the modality comparisons of language‐similarity between Korean‐English, Korean‐Chinese, and Chinese‐English to find any common regions showing higher language‐similarity in the auditory modality than the visual modality and vice versa.

We also calculated the similarity between the auditory and visual modalities for each language separately using one‐sample *t*‐tests. Then, using paired *t*‐tests, we compared the modality‐similarity between each pair of languages (i.e., Korean vs. Chinese, Korean vs. English, Chinese vs. English) with individual accuracy differences between the visual and auditory tasks as a covariate. A conjunction analysis was conducted between Chinese > Korean and English > Korean to reveal common regions that showed greater modality‐similarity for the L2s (Chinese and English) than for the L1 (Korean).

To compare language‐similarity between the language pairs (i.e., Korean‐Chinese vs. Korean‐English, Korean‐Chinese vs. Chinese‐English, Korean‐English vs. Chinese‐English) in each modality, we performed a series of paired *t*‐tests. We then conducted conjunction analyses to reveal whether any common regions showed higher similarity between the two L2s (Chinese and English) than between each L2 and L1 (Korean and Chinese, Korean, and English) in each modality. A threshold of uncorrected *p* < .001 was applied at the voxel level, and a threshold of the false discovery rate (FDR) corrected *p* < .05 was applied at the cluster level for all *t*‐tests and conjunction analyses.

## 3 RESULTS

### 3.1 Behavioral performance

The accuracy and reaction time on the task for each modality in each language are reported in Table 3. A 2 modality (visual vs. auditory) × 3 language (Korean vs. Chinese vs. English) repeated‐measure ANOVAs were conducted separately for accuracy and reaction time (RT). For accuracy, the main effect of modality was not significant [*F*_(1,21)_ = .358, *p* = .556], but the main effect of language was significant [*F*_(2,42)_ = 147.767, *p* < .001], with higher accuracy for Korean than for Chinese [*t*(21) = 8.714, *p* < .001] and English [*t*(21) = 16.513, *p* < .001]. The accuracy for Chinese was higher than that for English [*t*(21) = 8.880, *p* < .001]. In addition, the interaction between language and modality was significant [*F*_(2,42)_ = 42.036, *p* < .001]. Posthoc analyses revealed that the Korean task in the visual modality showed a higher accuracy than the Chinese task [*t*(21) = 10.097, *p* < .001] and the English task [*t*(21) = 20.305, *p* < .001]. The Chinese task had a higher accuracy than the English task [*t*(21) = 5.444, *p* < .001]. For the auditory modality, the Korean task showed a higher accuracy than the English task [*t*(21) = 5.398, *p* < .001], but not the Chinese task [*t*(21) = .797, *p* = .435]. The Chinese task showed a higher accuracy than the English task [*t*(21) = 8.287, *p* < .001].

**TABLE 3:** Means and standard deviations of age and behavioral performance in each language

		Korean				
		O + P+	O + P−	O − P+	O − P−	Overall
Accuracy (%)	Auditory	80.4 (10.7)	NA	72.3 (14.6)	96.1 (5.9)	82.9 (8.9)
	Visual	98.3 (3.5)	NA	96.8 (3.8)	97.6 (3.0)	97.5 (2.8)
RT (ms)	Auditory	1,368 (245)	NA	1,439 (200)	1,344 (225)	1,397 (195)
	Visual	999 (246)	NA	1,072 (258)	997 (234)	1,033 (262)

		English				
		O + P+	O + P−	O − P+	O − P−	
Accuracy (%)	Auditory	81.3 (8.8)	52.9 (11.3)	82.8 (11.2)	76.3 (12.7)	73.1 (6.0)
	Visual	87.4 (9.6)	28.6 (16.4)	68.9 (14.4)	83.4 (13.1)	67.1 (8.3)
RT (ms)	Auditory	1,361 (231)	1,442 (251)	1,386 (244)	1,378 (218)	1,392 (220)
	Visual	1,241 (218)	1,316 (264)	1,330 (280)	1,284 (268)	1,293 (258)

		Chinese				
		O + P+	O + P‐	O‐P+	O‐P‐	
Accuracy (%)	Auditory	70.8 (12.3)	92.6 (4.3)	78.7 (8.8)	95.9 (5.9)	84.2 (5.9)
	Visual	75.2 (11.5)	71.6 (18.3)	75.6 (11.2)	93.2 (12.7)	78.7 (9.4)
RT (ms)	Auditory	1,446 (196)	1,467 (241)	1,482 (209)	1,366 (183)	1,440 (188)
	Visual	1,308 (252)	1,365 (281)	1,352 (246)	1,248 (280)	1,319 (251)

In the RT analyses, there was a significant main effect of modality and language [*F*_(1,21)_ = 33.844, *p* < .001 for modality and *F*_(1,21)_ = 18.124, *p* < .001 for language]. The auditory modality had a longer RT than the visual modality. Korean had a shorter RT than Chinese [*t*(21) = 5.899, *p* < .001] and English [*t*(21) = 4.377, *p* < .001], but there was no significant difference between Chinese and English [*t*(21) = 1.271, *p* = .653]. In addition, the interaction between modality and language was significant [*F*_(2,42)_ = 11.48, *p* < .001]. Posthoc tests revealed that in the visual modality, the Korean task elicited faster RTs than the Chinese task [*t*(21) = 6.134, *p* < .001] and the English task [*t*(21) = 4.606, *p* < .001]. However, there was no significant difference in RT between the Chinese and English tasks (*t*(21) = .490, *p* = .629). In the auditory modality, there was no significant difference between any two languages [*t*(21) = 1.900, *p* = .071 for Korean and Chinese; *t*(21) = .150, *p* = .882 for Korean and English; *t*(21) = 1.681, *p* = .108 for Chinese and English].

### 3.2 Brain results

#### 3.2.1 Language‐similarity in each modality

**FIGURE 1:**
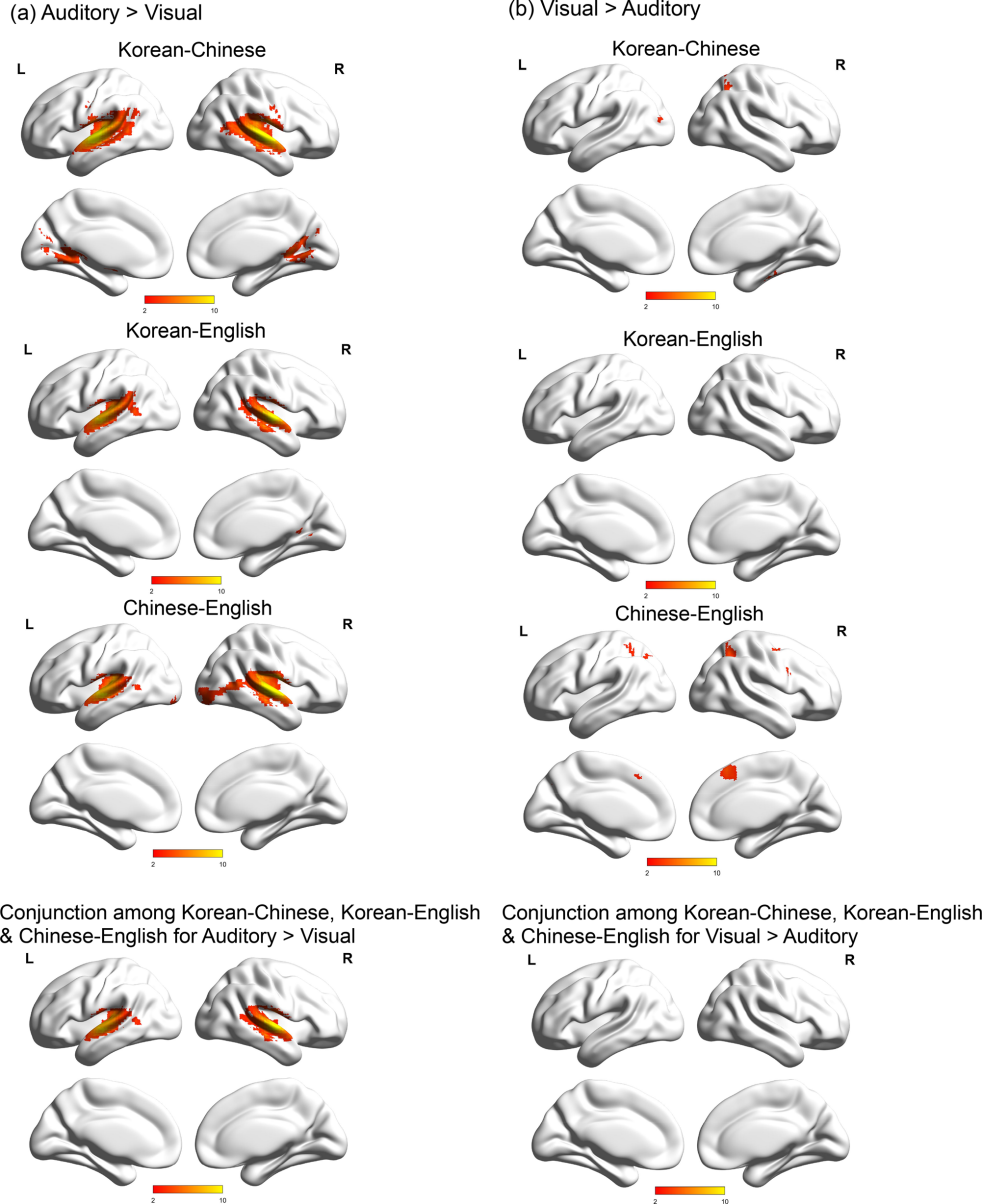
(a) Greater language‐similarity in the auditory modality than in the visual modality and (b) greater language‐similarity in the visual modality than in the auditory modality

We found significant PS between Korean and Chinese, between Korean and English, and between Chinese and English separately for the auditory and visual modalities in several bilateral cortical areas (Figure S1 and Table S1). For each pair of languages, we compared the language‐similarity in the two modalities (Figure 1 and Table 4). First, for the pair of Korean‐Chinese, greater language‐similarity for the auditory task than for the visual task was found in the bilateral superior temporal gyri (including Heschl's gyrus), left precentral gyrus, and right posterior cingulate. In contrast, greater language‐similarity for the visual modality than the auditory modality was found in the left middle temporal gyrus, precuneus, and right parahippocampal gyrus. For the pair of Korean‐English, greater language‐similarity for the auditory modality than the visual modality was found in the bilateral superior temporal gyri and right posterior cingulate, but no greater language‐similarity for the visual modality than the auditory modality was found. Last, for the pair of Chinese‐English, greater language‐similarity for the auditory modality than for the visual modality was found in the bilateral superior temporal gyri, left middle temporal gyrus, and lingual gyrus. Greater language‐similarity for the visual than the auditory modality was found in the left precuneus and right superior frontal gyrus.

**TABLE 4:** Comparisons between the modalities for the language‐similarity

Anatomical region	H	BA	Voxels	*x*	*y*	*z*	Z
Auditory ≥ visual							
*Korean*‐*Chinese*							
Superior temporal gyrus	R	22	1873	63	−9	3	Inf
	L	22	1,688	−48	−21	3	Inf
Posterior cingulate	R	30	998	6	−57	6	5.71
Precentral gyrus	L	6	25	−60	−9	36	4.08
*Korean*‐*English*							
Superior temporal gyrus	R	22	1,277	63	−9	3	Inf
	L	22	1,295	−48	−24	6	Inf
Posterior cingulate	R	30	63	6	−54	3	4.24
*Chinese*‐*English*							
Superior temporal gyrus	R	22	1878	63	−9	3	Inf
	L	22	1,171	−48	−21	3	Inf
Middle temporal gyrus	L	22	22	−54	−51	6	3.65
Lingual gyrus	L	18	39	−27	−87	−9	3.91
Conjunction among Korean‐Chinese, Korean‐English, & Chinese‐English for auditory ≥ visual							
Superior temporal gyrus	R	22	1,283	63	−9	3	Inf
	L	22	1,103	−48	−24	3	Inf
Visual ≥ auditory							
*Korean*‐*Chinese*							
Middle temporal gyrus	L	31	50	−33	−78	15	4.39
Parahippocampal gyrus	R	–	52	36	−24	−21	4.25
Precuneus	L	7	57	−18	−63	42	4.10
*Korean*‐*English*							
—							
*Chinese*‐*English*							
Precuneus	L	7	421	−18	−54	45	5.39
Superior frontal gyrus	R	8	79	6	24	54	4.38
Conjunction for Korean‐Chinese, Korean‐English, & Chinese‐English for visual ≥ auditory							
—	—						

The conjunction analyses of greater language‐similarity for the auditory modality than the visual modality between Korean and Chinese, between Korean and English, and between Chinese and English showed greater language‐similarity in the auditory modality than the visual modality in the bilateral superior temporal cortex (Figure 1 and Table 4). The conjunction analyses of visual greater than auditory for the language similarity between Korean and Chinese, between Korean and English, and between Chinese and English did not show any regions with greater language‐similarity in the visual modality than the auditory modality.

#### 3.2.2 Modality‐similarity within each language

For Korean and English, significant PS between the auditory and visual modalities was found, mostly in the left hemisphere, and for Chinese, significant PS between the modalities was found in bilateral cortical areas. All three languages consistently showed strong PS between modalities in the left inferior frontal gyrus (Figure S2 and Table S2).

**FIGURE 2:**
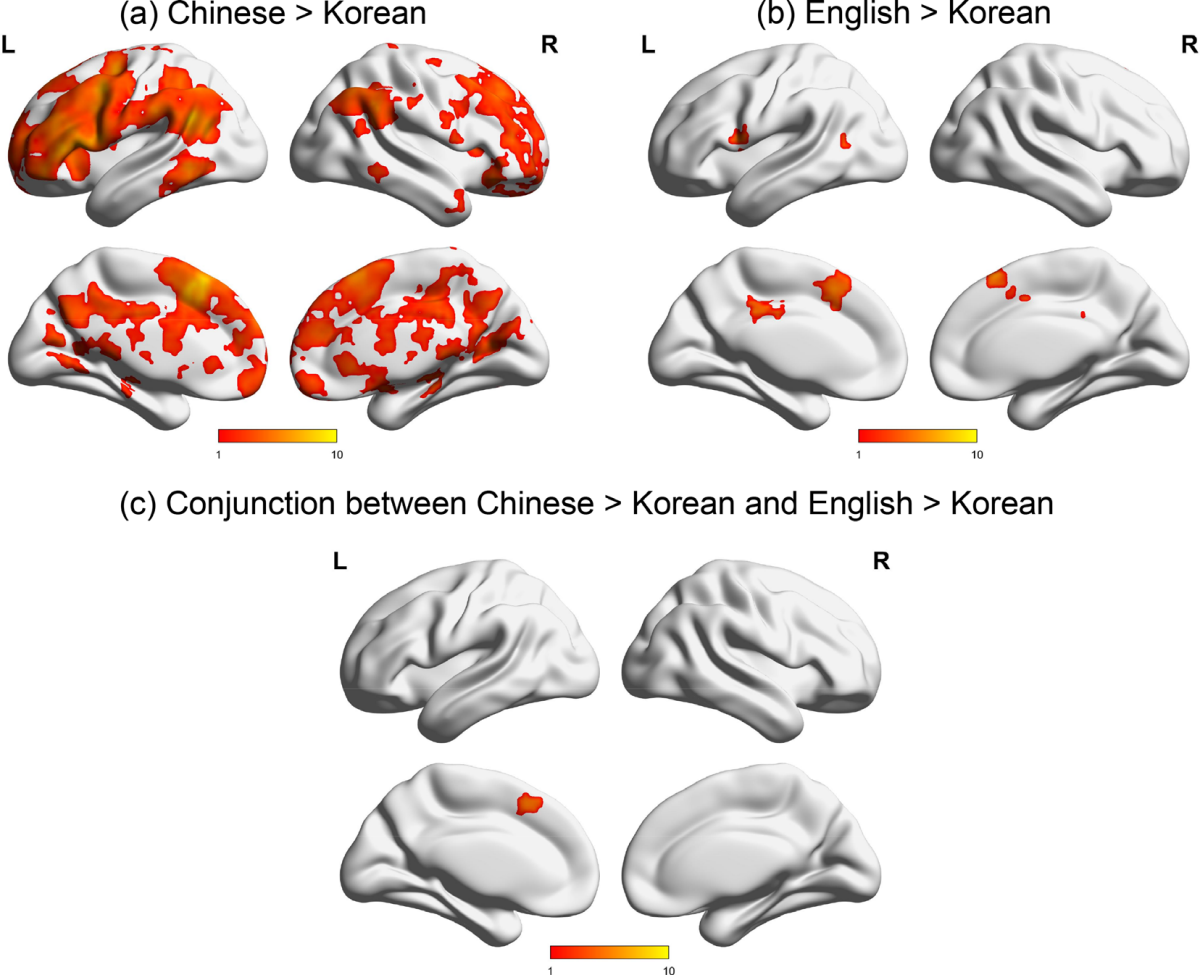
Greater modality‐similarity in L2s than L1. (a) Brain regions that showed greater modalitysimilarity in Chinse than in Korean; (b) brain regions that showed greater modality‐similarity in English than in Korean; (c) the conjunction between (a) and (b). No brain regions showed greater modality‐similarity in Korean than in Chinese or English

The language comparison results for modality‐similarity are presented in Figure 2 and Table 5. There was greater modality‐similarity in Chinese than Korean in the bilateral posterior cingulate, left supramarginal gyrus, medial frontal gyrus, middle temporal gyrus, middle/superior frontal gyrus, and right inferior frontal gyrus. There was greater modality‐similarity in English than Korean in the bilateral middle temporal gyri, left cingulate gyrus, pre/postcentral gyri, right superior frontal gyrus and inferior parietal lobule. Korean did not show greater modality‐similarity than Chinese or English at any region. There was no significant difference in modality‐similarity between the two L2s (Chinese and English). The conjunction analysis revealed that the left medial frontal gyrus consistently showed significantly greater modality‐similarity in Chinese and English than Korean.

**TABLE 5:** Comparisons between languages for the modality‐similarity

Anatomical region	H	BA	Voxels	*x*	*y*	*z*	Z
*Chinese ≥ Korean*							
Supramarginal gyrus	L	40	3,035	−45	−51	30	5.70
Medial frontal gyrus	L	8	781	−6	24	51	5.52
Posterior cingulate	L	30	162	−3	−54	3	4.81
	R	31	38	18	−69	12	4.22
Middle temporal gyrus	L	21	110	−57	−48	−3	4.55
Inferior frontal gyrus	R	9	41	57	12	36	4.28
Superior frontal gyrus	L	10	41	−6	66	−9	4.20
Precuneus	L	7	259	−6	−42	45	4.02
Middle frontal gyrus	R	11	21	15	42	−21	4.01
Inferior frontal gyrus	R	11	25	39	30	−15	3.94
*English ≥ Korean*							
Superior frontal gyrus	R	8	275	6	24	57	4.72
Cingulate gyrus	L	31	111	−3	−33	33	4.64
Middle temporal gyrus	L	22	23	−57	−63	12	4.04
*Conjunction between Chinese ≥ Korean and English ≥ Korean*							
Medial frontal gyrus	L	6	28	−6	18	48	4.72
*Korean ≥ Chinese*	—						
—							
*Korean ≥ English*	—						
—							
*English ≥ Chinese*	—						
—							

#### 3.2.3 Language‐similarity between L1 and L2

**FIGURE 3:**
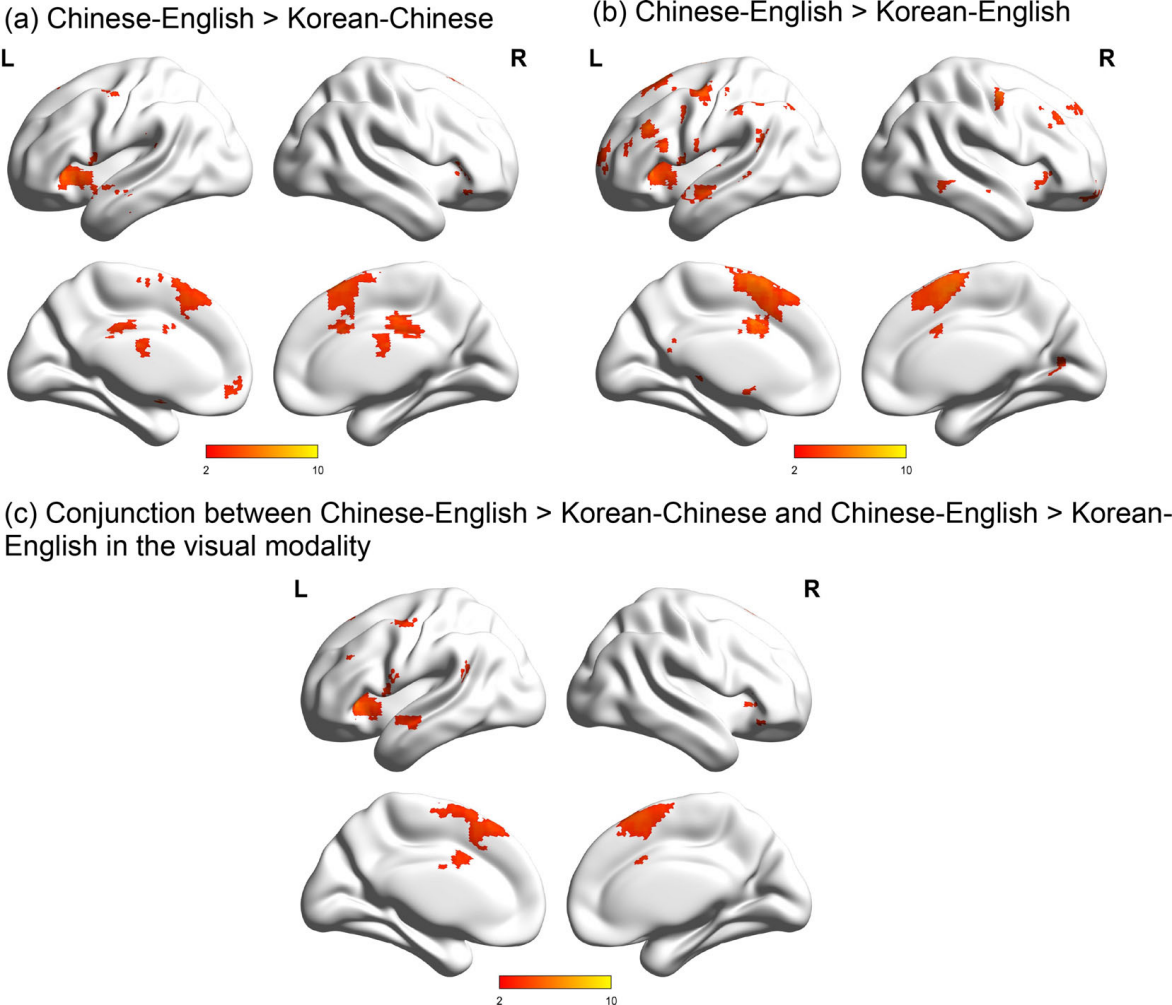
Greater language‐similarity between the two L2s than that between the L1 and each L2 in the visual task. (a) Brain regions that showed greater language‐similarity between the two L2s than that between Korean and Chinese; (b) brain regions that showed greater language‐similarity between the two L2s than that between Korean and English; (c) the conjunction between (a) and (b). No brain regions showed greater language‐similarity between the L1and L2 than that between the two L2s

We tested whether language‐similarity between the two L2s (i.e., Chinese and English) differs from that between L1 and L2 (either Korean‐Chinese or Korean‐English) separately for the visual and auditory modalities. In the visual modality, the similarity between the two L2s (Chinese‐English) was greater than that between Korean L1 and Chinese L2 in bilateral inferior frontal gyri, left inferior parietal lobule, middle temporal gyrus, precentral gyrus, medial frontal gyrus and right cingulate gyrus (Figure 3 and Table 6). The similarity between the two L2s (Chinese‐English) was also greater than that between Korean L1 and English L2 in the bilateral middle temporal gyri, precentral gyri, left middle frontal gyrus, supramarginal gyrus, and right superior frontal gyri. The conjunction analysis of greater similarity between Chinese‐English than that between Korean‐Chinese and greater similarity between Chinese‐English than that between Korean‐English revealed greater similarity between the two L2s than that between L1 and either L2 in several regions including the left precentral gyrus, middle frontal gyrus, middle temporal gyrus, inferior parietal lobule, and right inferior/superior frontal gyri showed. Neither Korean‐Chinese nor Korean‐English showed greater similarity than Chinese‐English at any region in the brain. In the auditory modality, we did not find significant differences between the L2 language‐similarity (i.e., Chinese and English) and L1‐L2 similarity (either Korean‐Chinese or Korean‐English).

**TABLE 6:** Comparisons of language‐similarity

Anatomical region	H	BA	Voxels	x	y	z	Z
Visual							
*Chinese*‐*English ≥ Korean*‐*Chinese*							
Inferior frontal gyrus	L	47	282	−33	27	−3	4.76
	R	45	64	51	24	6	4.17
Inferior parietal lobule	L	40	29	−57	−30	21	4.34
Cingulate gyrus	R	24	147	6	−18	39	4.33
Middle temporal gyrus	L	22	20	−60	−3	−9	3.72
Precentral gyrus	L	4	46	−45	−12	54	3.71
Medial frontal gyrus	L	10	36	−6	54	−6	3.69
*Korean*‐*Chinese ≥ Chinese*‐*English*							
—							
*Chinese*‐*English ≥ Korean*‐*English*							
Cingulate gyrus	–	32	978	0	12	−36	5.88
Middle frontal gyrus	L	9	111	−45	33	33	5.31
Precentral gyrus	L	4	291	−45	−12	54	5.10
	R	6	46	51	−9	54	4.67
Middle temporal gyrus	L	21	20	−54	−39	−6	4.17
	L	21	99	−54	−6	−12	4.17
Superior frontal gyrus	R	8	32	12	48	45	4.02
	R	9	70	39	33	36	4.01
	R	11	31	21	51	−15	3.96
Supramarginal gyrus	L	40	52	−57	−48	24	3.92
Middle temporal gyrus	R	21	28	60	−48	−9	3.82
Middle frontal gyrus	L	10	68	−24	66	9	3.79
*Korean*‐*English ≥ Chinese*‐*English*							
—							
Conjunction between Chinese‐English ≥ Korean‐Chinese & Chinese‐English ≥ Korean‐English							
Superior frontal gyrus	R	6	657	3	15	60	4.96
Precentral gyrus	L	6	94	−45	−12	54	4.32
	L	6	29	−54	6	9	3.68
Middle frontal gyrus	L	9	21	−36	30	30	4.11
Inferior frontal gyrus	R	45	76	51	24	3	4.03
Middle temporal gyrus	L	21	51	−60	−3	−9	3.91
Posterior cingulate	L	23	30	−3	−57	12	3.84
Inferior parietal lobule	L	40	35	−63	−45	21	3.81
*Korean*‐*Chinese ≥ Korean*‐*English*							
Middle occipital gyrus	R	18,19	276	21	−78	9	4.00
*Korean*‐*English ≥ Korean*‐*Chinese*							
—							
Auditory							
*Chinese*‐*English ≥ Korean*‐*Chinese*							
—							
*Korean*‐*Chinese ≥ Chinese*‐*English*							
—							
*Chinese*‐*English ≥ Korean*‐*English*							
—							
*Korean*‐*English ≥ Chinese*‐*English*							
*—*							
Conjunction between *Chinese*‐*English ≥ Korean*‐*Chinese & Chinese*‐*English ≥ Korean*‐*English*							
*—*							
*Korean*‐*Chinese ≥ Korean*‐*English*							
Angular gyrus	L	39	23	−42	−75	30	4.24
*Korean*‐*English ≥ Korean*‐*Chinese*							
—							

We also tested whether language‐similarity between Korean‐Chinese is different from that between Korean‐English in each modality. This analysis revealed greater language‐similarity between Korean and Chinese than that between Korean and English in the right middle occipital gyrus in the visual modality and in the left angular gyrus in the auditory modality (Table 6). No regions showed greater language‐similarity between Korean and English than that between Korean and Chinese in either modality.

## 4 DISCUSSION

In the present study, we examined how written and spoken words in L1 and L2s are processed similarly or differently in Korean‐English‐Chinese trilinguals using RSA. Spoken words appear to be processed more similarly in the three languages than written words, suggesting greater overlap in the network for spoken languages. More specifically, written words and spoken words seem to be processed more similarly in the two L2s than in L1, suggesting greater differentiation between spoken and written language processing in the native language. We also found that the two L2s are processed more similarly to each other than either of them is to the L1, especially for written words, suggesting that there might be an L2 network in the brain that could reflect accommodation to new writing systems after the L1 has been established in preferred brain areas. These findings are essential in understanding how spoken and written words in L1 and L2 are processed in the bilingual brain, thereby paving the way for understanding the brain mechanisms of reading disability in bilingual populations.

### 4.1 Greater language‐similarity in the auditory modality than in the visual modality

We found that, in the bilateral superior temporal regions, spoken words are processed more similarly in the three languages than written words. In the auditory task, many regions including the superior temporal gyrus showed a language‐similarity effect (Figure S1a). This provides evidence for a language‐universal network for spoken word processing that probably supports both basic sensory and language processing, which is consistent with a previous study (Rueckl et al., 2015). Spoken language processing is an essential skill in human communication with different designated brain regions, for example, Wernicke's area for listening comprehension (Binder, 2015), which is universal across languages and cultures (Bellugi, Poizner, & Klima, 1989).

Because reading is a relatively new cognitive function in human evolution, no brain regions are initially dedicated to reading. Instead, reading is supported by brain regions originally engaged in other functions, as proposed by the *neuronal recycling hypothesis* (Dehaene & Cohen, 2007) or *neuroemergentism* (Hernandez et al., 2019, for review). The diversity of visual forms and mapping rules across languages might contribute to the reduced language similarity in the visual modality (Bolger et al., 2005; Tan et al., 2005). We found that no brain regions showed greater language‐similarity in the visual modality than in the auditory modality. One would expect greater similarity between languages at the visuo‐orthographic regions in the brain for the visual task than for the auditory task. However, the absence of such findings is due to the lack of similarity between Korean and English in the visuo‐orthographic regions during the visual task. This might be because the visual form of English contrasts with that of Korean. (Figure S1b). The language‐similarity in the visual task is mainly located in the left inferior frontal gyrus, which also shows language‐similarity in the auditory task. This is consistent with the finding of Rueckl et al. (2015), which showed universality of brain activity at the left inferior frontal gyrus during print and speech processing in four contrastive languages (English, Hebrew, Spanish, and Chinese). Taken together, their results and ours suggest the essential role of the left inferior frontal gyrus in language processing, which might be related to phonological processing (see more discussion below). However, Rueckl et al. (2015) also emphasized a converged universality of brain signature for both spoken and written language, which differs from our emphasis on the direct contrast of spoken and written language. Taken together, for the rhyming task, we found commonality across languages in the left inferior frontal gyrus for both the auditory modality and the visual modality. We also found greater language similarity in the auditory modality than the visual modality in the superior temporal gyrus, suggesting a universal speech mechanism.

### 4.2 Greater modality‐similarity in L2s than L1

Spoken words and written words are processed similarly at the left inferior frontal gyrus in all three languages (−51, 6, 33 for Korean, −51, 18, 30 for Chinese, −54, 12, 12 for English). This suggests that the left inferior frontal gyrus is an important language‐core area independent of modality or language, which is consistent with two previous studies (Regev et al., 2013; Rueckl et al., 2015). The posterior inferior frontal gyrus has been shown to function in phonological working memory (Perrachione, Ghosh, Ostrovskaya, Gabrieli, & Kovelman, 2017; Vigneau et al., 2006), phonological retrieval (Costafreda et al., 2006; Katzev, Tüscher, Hennig, Weiller, & Kaller, 2013), and phonological manipulation (Booth, Bebko, Burman, & Bitan, 2007; Georgiewa et al., 1999), whereas the anterior inferior frontal gyrus is involved in semantic processing (Devlin, Matthews, & Rushworth, 2003; Gough, Nobre, & Devlin, 2005; Poldrack et al., 1999; Snyder, Feigenson, & Thompson‐Schill, 2007). Our study provides further evidence that the representational patterns of phonology from different languages in different modalities are similar in this region, suggesting its essential role in language processing.

We also found greater similarity between spoken and written word processing in Chinese than in Korean in a network including the left supramarginal gyrus and middle temporal gyrus, and greater similarity between spoken and written word processing in English than in Korean at the left middle temporal gyrus and right superior frontal gyrus. These results suggest that neural activity patterns are more specialized for spoken and written words in L1 than L2, which is consistent with previous findings that higher language proficiency is related to greater neural specialization (Debska et al., 2016; Van de Putte, De Baene, Brass, & Duyck, 2017; Zinszer, Chen, Wu, Shu, & Li, 2015). This is also consistent with a previous study conducted only in the native language that found dissociation between the visual and auditory modalities in the early‐stage visual and auditory regions and higher‐order parietal and frontal regions (Regev et al., 2013). This study found that the only overlapping region between the two modalities is in the superior temporal gyrus and inferior frontal gyrus, suggesting modality‐invariant linguistic processing in those regions. Furthermore, the conjunction analysis of Chinese > Korean and English > Korean revealed that the left medial frontal gyrus showed greater modality‐similarity for the two L2s than L1. This finding is consistent with a recent meta‐analysis study by Cargnelutti, Tomasino, and Fabbro (2019), which showed that the left medial frontal region (and inferior frontal region) is more consistently activated for L2 than L1, presumably due to the greater attentional and cognitive effort involved in processing L2 than L1. The medial frontal area is an important part of the attention network (e.g., Brown & Braver, 2005; Kearn et al., 2004; Schall, Shtuphorn, & Brown, 2002). Taken together, our findings indicate that spoken and written word processing evoke more similar activation patterns in a diffused network in L2s than in L1, suggesting less specialization. Among those regions, both Chinese and English showed greater similarity between spoken and written word processing in the left medial frontal gyrus than in the L1, Korean, which might be due to the more effortful processing of L2.

### 4.3 Greater language‐similarity in L2s

In the present study, we found that language‐similarity between the two L2s was greater than that between L1 and each of the L2s in the visual modality in the left middle frontal gyrus, precentral gyrus, middle temporal gyrus, inferior parietal lobule, posterior cingulate and right superior and inferior frontal gyri. These findings are unlikely to be related to more similar proficiency, task performance, or AOA in the two L2s than that in the L1 and L2, because (1) the performance was regressed out as a covariate in all data analyses, (2) the accuracy difference between Chinese and English was as great as that between Korean and Chinese in the visual task, and the accuracy difference between Chinese and English was greater than that between Korean and Chinese in the auditory task, and (3) Chinese had a higher proficiency and later AOA than English in the current study. Therefore, the greater similarity of representational patterns between the L2s in these important reading/language regions might support the critical period hypothesis (Lenneberg, 1967; Newport, 1990) that supposes that a language acquired later in life is represented and processed qualitatively differently from L1. Our findings add to the critical period hypothesis by showing that the neural commitment the brain makes is critical during its first exposures to language and the effect that commitment has on the exposures to later languages in recruiting different neural territory. Any language that is acquired after L1 (and after the critical period) might be processed in a different network than L1, and proficiency or AOA in the L2 only plays a limited role. Thus, there seems to be a greater difference between L1 and L2 than that driven by language difference, as proposed in Kim et al.'s study (2016). Kim et al. (2016) compared brain activation patterns between Korean L1 and two L2s (Chinese and English) and found the English L2 brain network is similar to the Korean L1 network but different from the native English network, whereas the Chinese L2 brain network is more similar to the native Chinese network than to the Korean L1 network. As a partial motivation of the present study, we calculated the similarity index between English L2 and Chinese L2 using data and formulae from Kim's 2016 study, and found that the similarity between Chinese and English is .958, which is higher than that between Korean and Chinese (.658), but lower than that between Korean and English (.999). Therefore, as we expected, the similarity between the two L2s was higher than some of the L1‐L2 similarity (i.e., Korean‐Chinese), however, higher similarity between Korean‐English than between Korean‐Chinese might be driven by language difference. The inconsistency between the two studies might be due to the different methods. Brain activation, which is based on a univariate approach, and representational similarity, which is based on a multivariate approach, may reflect different aspects of the brain activity.

Among the affected brain regions, the left inferior parietal lobule has been shown to be critical for learning L2 (Barbeau et al., 2017; Golestani & Zatorre, 2004), with the activation level of this region was significantly correlated with improvement in L2 (not L1) reading speed after 12 weeks of L2 training. This region also shows increased gray matter volume in bilinguals as compared with age‐matched monolinguals (Abutalebi, Canini, Della Rosa, Green, & Weekes, 2015), suggesting its special role in L2 acquisition. In addition, the left inferior parietal lobule is related to verbal working memory (Alain, He, & Grady, 2008), which could be why it is important in L2 learning.

The regions that showed greater similarity between the L2s also included those involved in resolving challenges in reading, such as inconsistent or irregular words reading. For instance, the left middle frontal gyrus has been found to be heavily involved in making rhyming judgment about inconsistent words (Binder, Medler, Desai, Conant, & Liebenthal, 2005; Bolger, Hornickel, Cone, Burman, & Booth, 2008; Fiez, Balota, Raichle, & Petersen, 1999; Katz et al., 2005). In addition, a previous meta‐analysis (Sebastian, Laird, & Kiran, 2011) interpreted the increased involvement of the right inferior frontal gyri as a possible compensation for low language proficiency in L2, which is consistent with previous findings of greater involvement of the right hemisphere in L2 in general (Meschyan & Hernandez, 2006; Yokoyama et al., 2006). Alternatively, the involvement of those regions in L2 might also be because they are acquired after the critical period, so different mechanisms were recruited compared to L1 acquisition, irrespective of proficiency. Our current data support this latter idea because the participants had different proficiency in the two L2s, but they still showed similar patterns in those regions.

Lastly, in the visual modality, we found a greater language‐similarity between Chinese and Korean than that between English and Korean in the right middle occipital gyrus. This is consistent with previous findings that the right middle occipital region is involved in visuo‐orthographic processing for visually complicated scripts such as Chinese and Korean, but not English (Cao et al., 2010; Kim et al., 2016). However, in contrast to the previous study, which found greater similarity between English and Korean than between Chinese and Korean in Korean‐English‐Chinese trilinguals during visual word rhyming judgment (Kim et al., 2016), we found greater similarity between Korean and Chinese than that between Korean and English, perhaps because the two studies used different fMRI analysis methods. Although the univariate approach found that English and Korean activated more common regions (Kim et al., 2016), our multivariate approach found that Chinese and Korean share a more similar activation pattern across multiple voxels.

### 4.4 Limitations

One limitation in the current study is that the materials were not perfectly matched in the three languages. Monosyllabic words were used in English. In contrast, two‐syllable words were used in Korean and Chinese on the assumption that monosyllabic words in these languages would activate homophones and cause ambiguity in meaning and orthography. In addition, as a transparent language, O + P− was missing in Korean. The absence of O + P− in Korean likely explains the overall better performance in the visual modality than in the auditory modality in Korean. However, previous research also showed greater performance on the visual rhyming judgment than the auditory judgment task for native Chinese speakers (Cao et al., 2010; Cao et al., 2011) and native English speakers (Booth et al., 2004), suggesting that it might be easier to make a rhyming judgment when the words are visually presented for native speakers. Taken together, there is a small possibility that the greater similarity between Chinese and English than that between L1 and L2 in the visual modality is due to the fact that there is O + P− in Chinese and English. However, we tend to believe that this is a general L2 effect rather than a specific effect driven by the current languages and tasks. Future research on other languages and tasks should be conducted.

Another limitation of the current study is the unmatched AOA and proficiency level in Chinese and English. The AOA is earlier in English than Chinese, whereas the proficiency is higher in Chinese than in English. However, we found Chinese and English to be similar instead of different in the current study. Specifically, we found (1) the modality‐similarity was comparable in Chinese and English, both of which were higher than Korean, (2) Chinese and English were similar in the visual task and both of them differed from Korean. Therefore, if AOA and proficiency had been better matched, our effects would be even stronger.

## 5 CONCLUSION

In the present study, greater language‐similarity across the three languages in the auditory modality than the visual modality was found. This is evidence for suggesting more differentiated network for written words than spoken words probably due to the salient diversity across orthographies. Less similarity between auditory and visual processing in L1 than L2s implies greater specialization for written and spoken word processing in L1. In addition, the similarity between the two L2s is generally greater than that between each L2 and L1, suggesting L2s might be represented and processed in a qualitatively different way than L1, in which AOA and proficiency may only play a limited role.

## CONFLICT OF INTEREST

The authors declare no conflict of interest.

## Supporting information


**Appendix** **S1:** Supplementary Information.

## Data Availability

The data that support the findings of this study are available from the corresponding author upon reasonable request. The full list of stimuli is also available based on request.
