# Supplementary material for: Neural representational similarity between L1 and L2 in spoken and written language processing
Source: Hum Brain Mapp. 2020 Aug 21;41(17):4935–51. doi: 10.1002/hbm.25171 (PMC7643388; doi:10.1002/hbm.25171)

Supplementary Table 1. Between-language PS in each modality

| Anatomical Region | H | BA | Voxels | x | y | z | Z |
|---|---|---|---|---|---|---|---|
| **Auditory** | | | | | | | |
| ***Korean-Chinese*** | | | | | | | |
| Superior temporal gyrus | R | 41 | 2930 | 60 | -18 | 6 | inf |
| | L | 22 | 5098 | -63 | -15 | 6 | inf |
| Posterior cingulate | L | 23 | 551 | -3 | -66 | 12 | 7.03 |
| Middle frontal gyrus | R | 9 | 120 | 45 | 12 | 30 | 6.76 |
| Lingual gyrus | L | 19 | 36 | -18 | -60 | -9 | 6.16 |
| ***Korean-English*** | | | | | | | |
| Superior temporal gyrus | L | 42 | 4818 | -66 | -27 | 12 | inf |
| | R | 22 | 2163 | 57 | -18 | 0 | inf |
| Superior frontal gyrus | L | 6 | 516 | -3 | 12 | 63 | 7.45 |
| Posterior cingulate | R | 29 | 161 | 9 | -51 | 6 | 6.55 |
| Superior frontal gyrus | L | 10 | 61 | -27 | 57 | 6 | 5.78 |
| | R | 10 | 23 | 27 | 60 | 0 | 5.57 |
| Precuneus | R | 19 | 74 | 36 | -78 | 39 | 5.50 |
| Precentral gyrus | R | 6 | 74 | 48 | -3 | 54 | 5.31 |
| ***Chinese-English*** | | | | | | | |
| Superior temporal gyrus | L/R | 22 | 11939 | 63 | -9 | 3 | inf |
| Superior frontal gyrus | L | 8 | 532 | -3 | 21 | 57 | inf |
| Inferior frontal gyrus | R | 9 | 257 | 54 | 9 | 38 | 6.99 |
| | R | 46 | 66 | 45 | 39 | 15 | 6.37 |
| Superior frontal gyrus | L | 8 | 70 | -27 | 39 | 48 | 6.25 |
| **Visual** | | | | | | | |
| ***Korean-Chinese*** | | | | | | | |
| Middle occipital gyrus | L/R | 19 | 8099 | -51 | -75 | 0 | inf |
| Middle frontal gyrus | L | 9 | 1652 | -48 | 15 | 33 | inf |
| | R | 9 | 365 | 48 | 12 | 30 | 6.40 |
| Cingulate gyrus | R | 31 | 46 | 3 | -39 | 33 | 6.28 |
| Middle temporal gyrus | R | 21 | 82 | 60 | -33 | -6 | 5.89 |
| ***Korean-English*** | | | | | | | |
| Inferior frontal gyrus | L | 9 | 560 | -51 | 6 | 33 | 7.80 |
| Inferior parietal lobule | L | 40 | 246 | -48 | -39 | 45 | 5.67 |
| ***Chinese-English*** | | | | | | | |
| Middle/Superior frontal gyrus | L | 9 | 11874 | -54 | 18 | 30 | Inf |
| Inferior frontal gyrus | R | 9 | 1427 | 48 | 9 | 30 | inf |
| Cuneus | L | 19 | 33 | -12 | -87 | 21 | 6.13 |
| | L | 18 | 30 | -3 | -93 | 15 | 5.99 |

For Peer Review

Supplementary Table 2. Between-modality PS in each language

| Anatomical Region | H | BA | Voxels | x | y | z | Z |
|---|---|---|---|---|---|---|---|
| ***Korean*** | | | | | | | |
| Inferior frontal gyrus | L | 9 | 7516 | -51 | 6 | 33 | inf |
| Middle temporal gyrus | R | 21 | 1454 | 51 | -57 | 3 | 5.24 |
| Middle frontal gyrus | R | 6 | 606 | 48 | 0 | 42 | 4.79 |
| Middle occipital gyrus | R | 18 | 59 | 24 | -96 | 0 | 4.40 |
| Superior frontal gyrus | R | 8 | 41 | 21 | 45 | 48 | 3.88 |
| ***Chinese*** | | | | | | | |
| Inferior/Middle frontal gyrus | L | 9 | 2170 | -51 | 18 | 30 | inf |
| Inferior parietal lobule | L | 40 | 1161 | -57 | -36 | 39 | 7.68 |
| | R | 40 | 440 | 42 | -66 | 39 | 6.48 |
| Middle frontal gyrus | R | 8 | 304 | 39 | 27 | 48 | 5.91 |
| | R | 46 | 110 | 54 | 36 | 18 | 5.86 |
| | R | 10 | 38 | 36 | 57 | 0 | 5.42 |
| ***English*** | | | | | | | |
| Inferior frontal gyrus | L | 44/46 | 2069 | -54 | 12 | 12 | 7.11 |
| Middle temporal gyrus | L | 21 | 1217 | -54 | -57 | 3 | 6.79 |
| | R | 21 | 36 | 60 | -48 | -6 | 6.07 |
| Superior temporal gyrus | R | 22 | 224 | 63 | -42 | 18 | 6.42 |
| Cingulate gyrus | L | 31 | 164 | -6 | -39 | 39 | 5.89 |
| Precuneus | L | 19 | 68 | -3 | -75 | 39 | 5.46 |
| Posterior cingulate | R | 29 | 23 | 9 | -54 | 6 | 5.25 |

For Peer Review

Supplementary Table 3. Conjunction analysis of between-language PS in the two modalities

| Anatomical Region | H | BA | Voxels | x | y | z | Z |
|---|---|---|---|---|---|---|---|
| **Conjunction analysis between the visual and auditory modality** | | | | | | | |
| ***Korean-Chinese*** | | | | | | | |
| Middle frontal gyrus | L | 9 | 1416 | -48 | 15 | 33 | inf |
| Middle occipital gyrus | L | 37 | 5247 | -54 | -72 | 0 | inf |
| Inferior frontal gyrus | R | 9 | 280 | 48 | 12 | 30 | 6.39 |
| Middle temporal gyrus | R | 21 | 74 | 60 | -33 | -6 | 5.89 |
| Cingulate gyrus | R | 31 | 36 | 6 | -39 | 33 | 5.84 |
| ***Korean-English*** | | | | | | | |
| Inferior frontal gyrus | L | 9 | 558 | -51 | 6 | 33 | 7.69 |
| Superior parietal lobule | L | 7 | 81 | -27 | -57 | 48 | 5.67 |
| Inferior parietal lobule | L | 40 | 51 | -54 | -42 | 51 | 5.55 |
| ***Chinese-English*** | | | | | | | |
| Middle frontal gyrus | L | 9 | 12560 | -54 | 18 | 30 | Inf |
| Superior frontal gyrus | L | 8 | 794 | -3 | 21 | 57 | inf |
| Inferior frontal gyrus | R | 9 | 845 | 54 | 9 | 36 | 6.99 |

For Peer Review

Supplementary Figure 1. Language-Similarity: (a) Auditory, (b) Visual, (c) Conjunction between the modalities

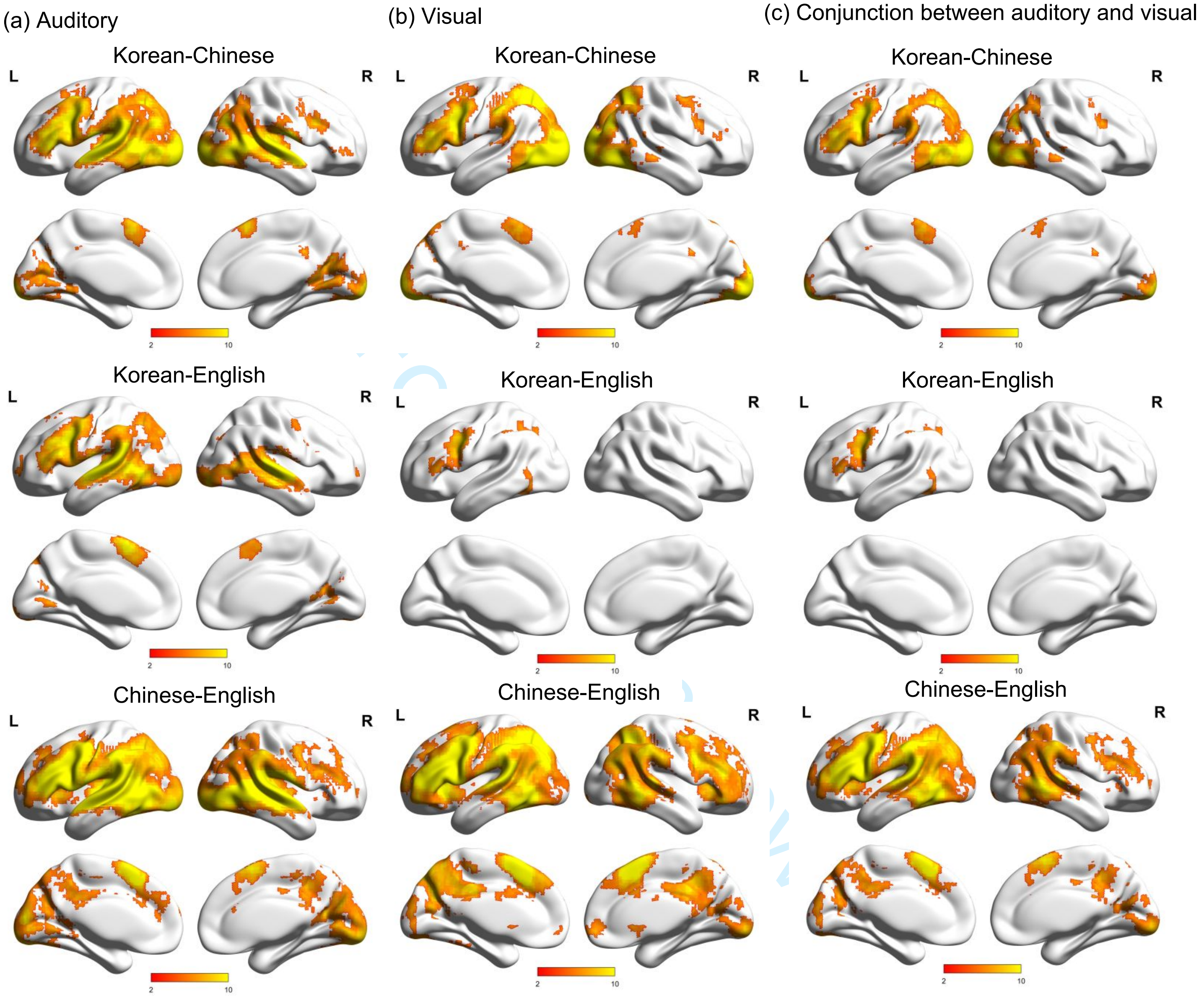

Supplementary Figure 2. Modality-similarity in each language

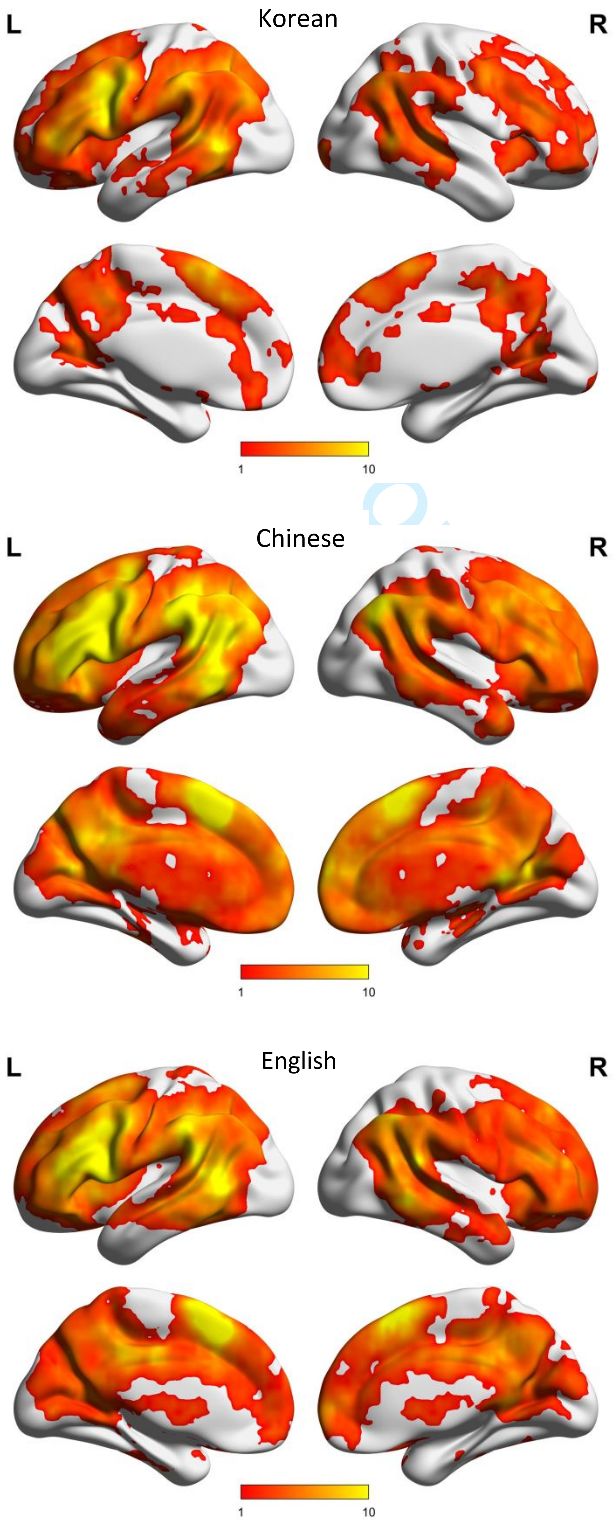

Supplement: Supplementary file 1 — Appendix S1: Supplementary Information. [file HBM-41-4935-s001.pdf]
